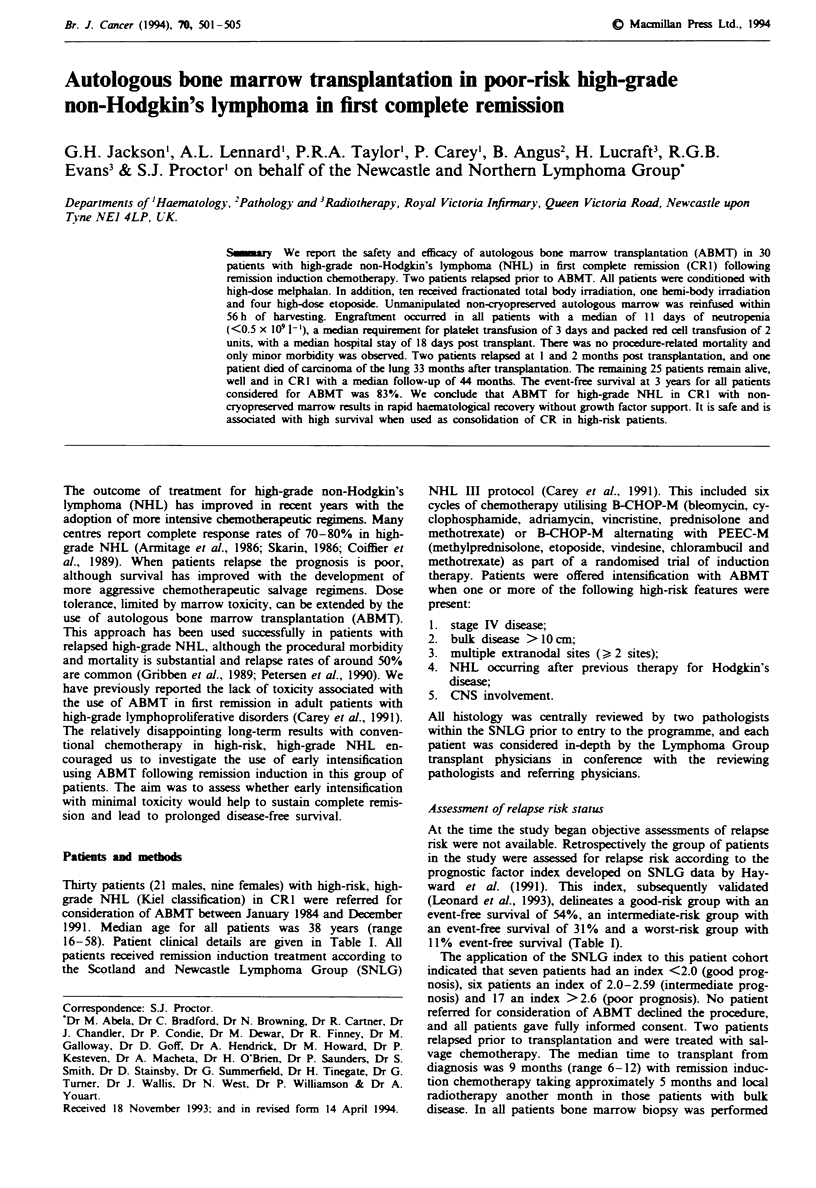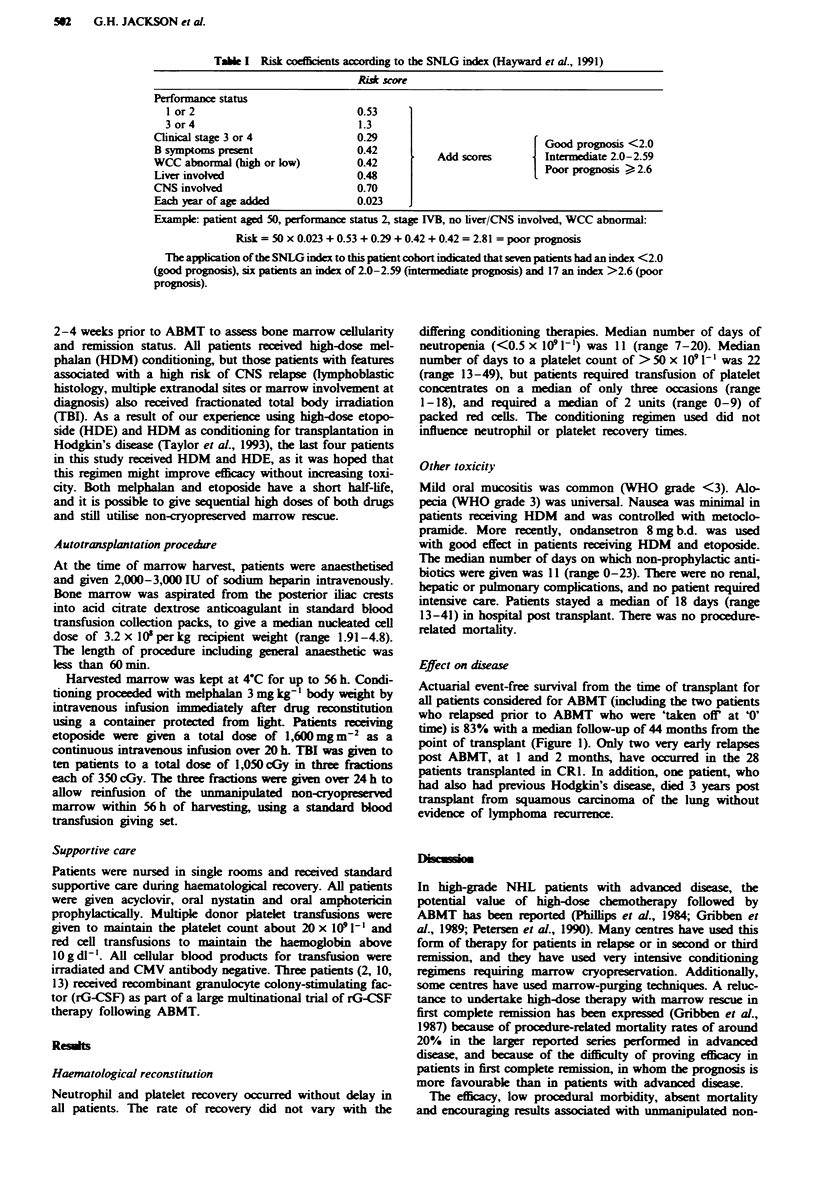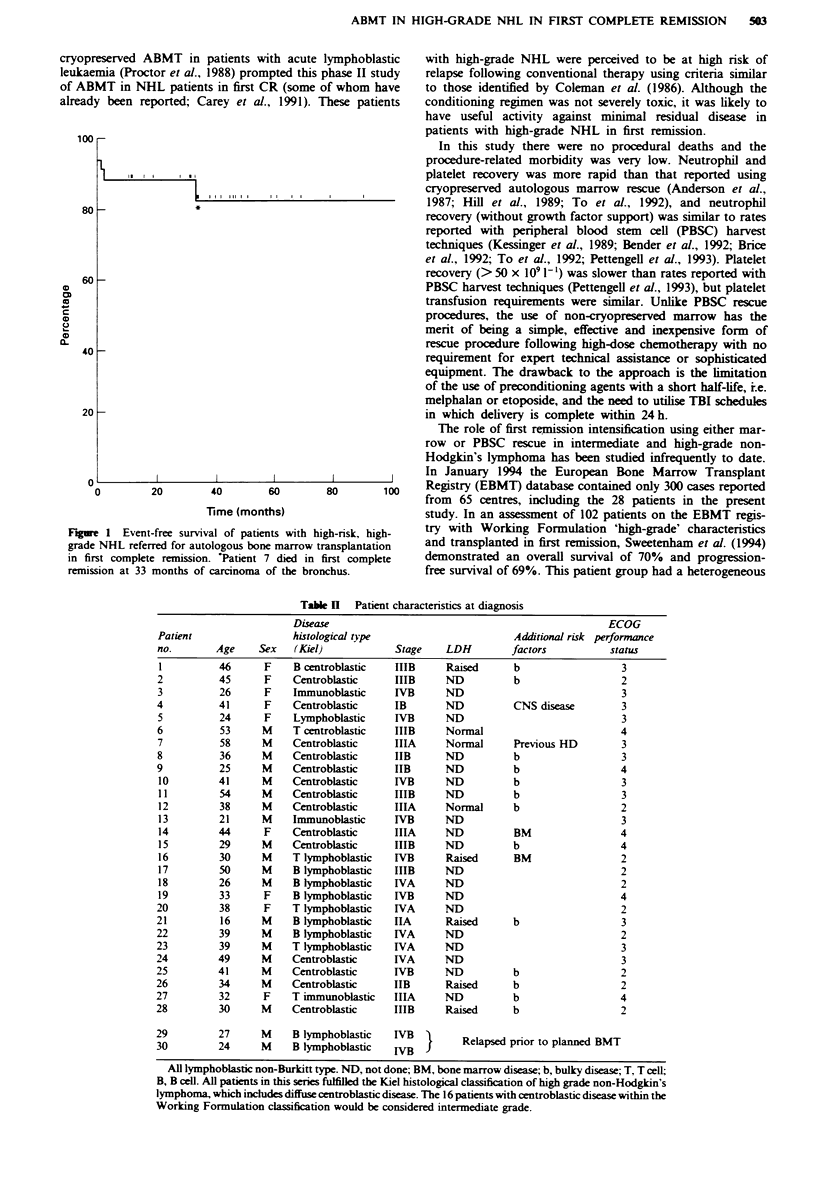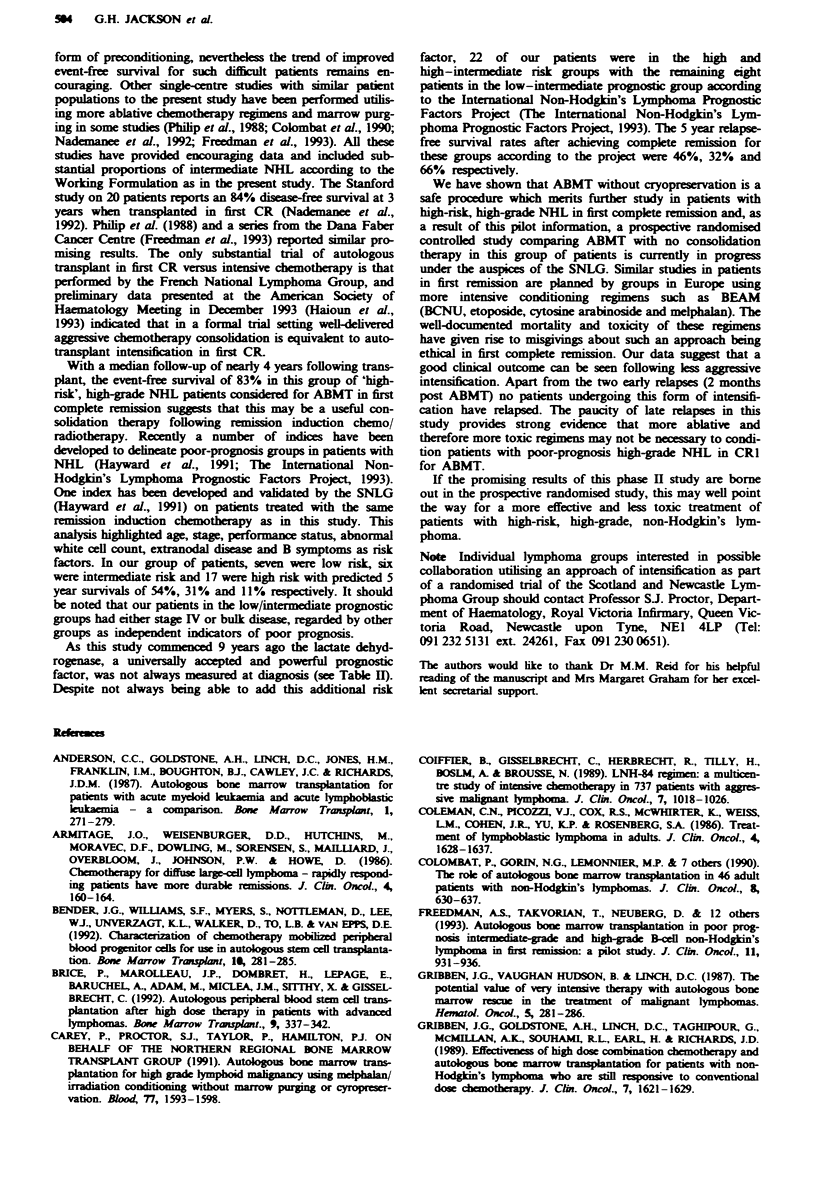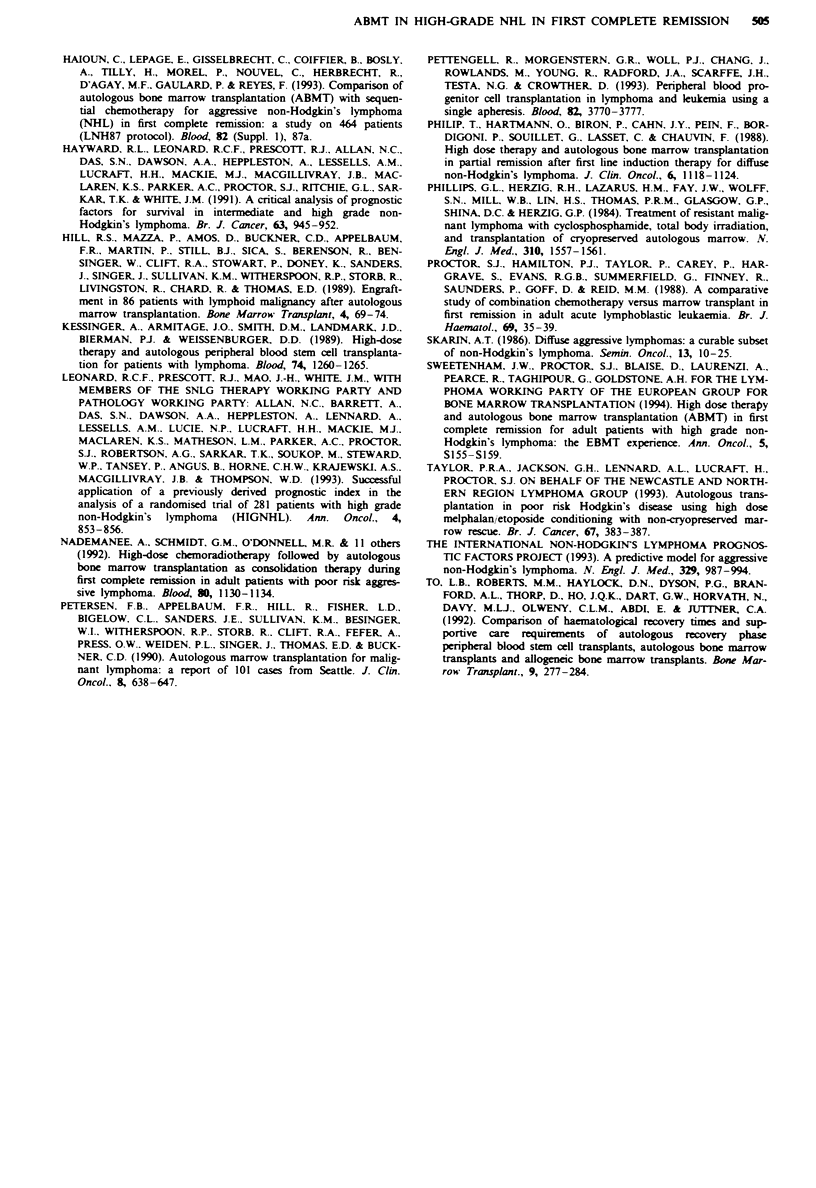# Autologous bone marrow transplantation in poor-risk high-grade non-Hodgkin's lymphoma in first complete remission. Newcastle and Northern Lymphoma Group.

**DOI:** 10.1038/bjc.1994.335

**Published:** 1994-09

**Authors:** G. H. Jackson, A. L. Lennard, P. R. Taylor, P. Carey, B. Angus, H. Lucraft, R. G. Evans, S. J. Proctor

**Affiliations:** Department of Haematology, Royal Victoria Infirmary, Newcastle upon Tyne, UK.

## Abstract

We report the safety and efficacy of autologous bone marrow transplantation (ABMT) in 30 patients with high-grade non-Hodgkin's lymphoma (NHL) in first complete remission (CR1) following remission induction chemotherapy. Two patients relapsed prior to ABMT. All patients were conditioned with high-dose melphalan. In Addition, ten received fractionated total body irradiation, one hemi-body irradiation and four high-dose etoposide. Unmanipulated non-cryopreserved autologous marrow was reinfused within 56 h of harvesting. Engraftment occurred in all patients with a median of 11 days of neutropenia (< 0.5 x 10(9) l-1), a median requirement for platelet transfusion of 3 days and packed red cell transfusion of 2 units, with a median hospital stay of 18 days post transplant. There was no procedure-related mortality and only minor morbidity was observed. Two patients relapsed at 1 and 2 months post transplantation, and one patient died of carcinoma of the lung 33 months after transplantation. The remaining 25 patients remain alive, well and in CR1 with a median follow-up of 44 months. The event-free survival at 3 years for all patients considered for ABMT was 83%. We conclude that ABMT for high-grade NHL in CR1 with non-cryopreserved marrow results in rapid haematological recovery without growth factor support. It is safe and is associated with high survival when used as consolidation of CR in high-risk patients.


Br. J. Cancer (1994), 70, 501-505 © Macmillan Press Ltd., 1994

### Autologous bone marrow transplantation in poor-risk high-grade non-Hodgkin's lymphoma in first complete remission

G.H. Jackson[1], A.L. Lennard[1], P.R.A. Taylor[1], P. Carey[1], B. Angus[2], H. Lucraft[3], R.G.B. Evans[3] & S.J. Proctor[1] on behalf of the Newcastle and Northern Lymphoma Group*

*Departments of [1]Haematology, [2]Pathology and [3]Radiotherapy, Royal Victoria Infirmary, Queen Victoria Road, Newcastle upon Tyne NE1 4LP, UK.*

**Summary** We report the safety and efficacy of autologous bone marrow transplantation (ABMT) in 30 patients with high-grade non-Hodgkin's lymphoma (NHL) in first complete remission (CR1) following remission induction chemotherapy. Two patients relapsed prior to ABMT. All patients were conditioned with high-dose melphalan. In addition, ten received fractionated total body irradiation, one hemi-body irradiation and four high-dose etoposide. Unmanipulated non-cryopreserved autologous marrow was reinfused within 56 h of harvesting. Engraftment occurred in all patients with a median of 11 days of neutropenia ($<0.5 \times 10^9 l^{-1}$), a median requirement for platelet transfusion of 3 days and packed red cell transfusion of 2 units, with a median hospital stay of 18 days post transplant. There was no procedure-related mortality and only minor morbidity was observed. Two patients relapsed at 1 and 2 months post transplantation, and one patient died of carcinoma of the lung 33 months after transplantation. The remaining 25 patients remain alive, well and in CR1 with a median follow-up of 44 months. The event-free survival at 3 years for all patients considered for ABMT was 83%. We conclude that ABMT for high-grade NHL in CR1 with non-cryopreserved marrow results in rapid haematological recovery without growth factor support. It is safe and is associated with high survival when used as consolidation of CR in high-risk patients.

The outcome of treatment for high-grade non-Hodgkin's lymphoma (NHL) has improved in recent years with the adoption of more intensive chemotherapeutic regimens. Many centres report complete response rates of 70–80% in high-grade NHL (Armitage *et al.*, 1986; Skarin, 1986; Coiffier *et al.*, 1989). When patients relapse the prognosis is poor, although survival has improved with the development of more aggressive chemotherapeutic salvage regimens. Dose tolerance, limited by marrow toxicity, can be extended by the use of autologous bone marrow transplantation (ABMT). This approach has been used successfully in patients with relapsed high-grade NHL, although the procedural morbidity and mortality is substantial and relapse rates of around 50% are common (Gribben *et al.*, 1989; Petersen *et al.*, 1990). We have previously reported the lack of toxicity associated with the use of ABMT in first remission in adult patients with high-grade lymphoproliferative disorders (Carey *et al.*, 1991). The relatively disappointing long-term results with conventional chemotherapy in high-risk, high-grade NHL encouraged us to investigate the use of early intensification using ABMT following remission induction in this group of patients. The aim was to assess whether early intensification with minimal toxicity would help to sustain complete remission and lead to prolonged disease-free survival.

### Patients and methods

Thirty patients (21 males, nine females) with high-risk, high-grade NHL (Kiel classification) in CR1 were referred for consideration of ABMT between January 1984 and December 1991. Median age for all patients was 38 years (range 16–58). Patient clinical details are given in Table I. All patients received remission induction treatment according to the Scotland and Newcastle Lymphoma Group (SNLG) NHL III protocol (Carey *et al.*, 1991). This included six cycles of chemotherapy utilising B-CHOP-M (bleomycin, cyclophosphamide, adriamycin, vincristine, prednisolone and methotrexate) or B-CHOP-M alternating with PEEC-M (methylprednisolone, etoposide, vindesine, chlorambucil and methotrexate) as part of a randomised trial of induction therapy. Patients were offered intensification with ABMT when one or more of the following high-risk features were present:

1. stage IV disease;
2. bulk disease >10 cm;
3. multiple extranodal sites (⩾2 sites);
4. NHL occurring after previous therapy for Hodgkin's disease;
5. CNS involvement.

All histology was centrally reviewed by two pathologists within the SNLG prior to entry to the programme, and each patient was considered in-depth by the Lymphoma Group transplant physicians in conference with the reviewing pathologists and referring physicians.

### *Assessment of relapse risk status*

At the time the study began objective assessments of relapse risk were not available. Retrospectively the group of patients in the study were assessed for relapse risk according to the prognostic factor index developed on SNLG data by Hayward *et al.* (1991). This index, subsequently validated (Leonard *et al.*, 1993), delineates a good-risk group with an event-free survival of 54%, an intermediate-risk group with an event-free survival of 31% and a worst-risk group with 11% event-free survival (Table I).

The application of the SNLG index to this patient cohort indicated that seven patients had an index <2.0 (good prognosis), six patients an index of 2.0–2.59 (intermediate prognosis) and 17 an index >2.6 (poor prognosis). No patient referred for consideration of ABMT declined the procedure, and all patients gave fully informed consent. Two patients relapsed prior to transplantation and were treated with salvage chemotherapy. The median time to transplant from diagnosis was 9 months (range 6–12) with remission induction chemotherapy taking approximately 5 months and local radiotherapy another month in those patients with bulk disease. In all patients bone marrow biopsy was performed

Correspondence: S.J. Proctor.

*Dr M. Abela, Dr C. Bradford, Dr N. Browning, Dr R. Cartner, Dr J. Chandler, Dr P. Condie, Dr M. Dewar, Dr R. Finney, Dr M. Galloway, Dr D. Goff, Dr A. Hendrick, Dr M. Howard, Dr P. Kesteven, Dr A. Macheta, Dr H. O'Brien, Dr P. Saunders, Dr S. Smith, Dr D. Stainsby, Dr G. Summerfield, Dr H. Tinegate, Dr G. Turner, Dr J. Wallis, Dr N. West, Dr P. Williamson & Dr A. Youart.

Received 18 November 1993; and in revised form 14 April 1994.

**Table I** Risk coefficients according to the SNLG index (Hayward *et al.*, 1991)

| | *Risk score* | | |
|---|---|---|---|
| Performance status | | | |
| 1 or 2 | 0.53 | Add scores | Good prognosis <2.0 |
| 3 or 4 | 1.3 | | Intermediate 2.0–2.59 |
| Clinical stage 3 or 4 | 0.29 | | Poor prognosis ⩾2.6 |
| B symptoms present | 0.42 | | |
| WCC abnormal (high or low) | 0.42 | | |
| Liver involved | 0.48 | | |
| CNS involved | 0.70 | | |
| Each year of age added | 0.023 | | |

Example: patient aged 50, performance status 2, stage IVB, no liver/CNS involved, WCC abnormal:

$$\text{Risk} = 50 \times 0.023 + 0.53 + 0.29 + 0.42 + 0.42 = 2.81 = \text{poor prognosis}$$

The application of the SNLG index to this patient cohort indicated that seven patients had an index <2.0 (good prognosis), six patients an index of 2.0–2.59 (intermediate prognosis) and 17 an index >2.6 (poor prognosis).

2–4 weeks prior to ABMT to assess bone marrow cellularity and remission status. All patients received high-dose melphalan (HDM) conditioning, but those patients with features associated with a high risk of CNS relapse (lymphoblastic histology, multiple extranodal sites or marrow involvement at diagnosis) also received fractionated total body irradiation (TBI). As a result of our experience using high-dose etoposide (HDE) and HDM as conditioning for transplantation in Hodgkin's disease (Taylor *et al.*, 1993), the last four patients in this study received HDM and HDE, as it was hoped that this regimen might improve efficacy without increasing toxicity. Both melphalan and etoposide have a short half-life, and it is possible to give sequential high doses of both drugs and still utilise non-cryopreserved marrow rescue.

### *Autotransplantation procedure*

At the time of marrow harvest, patients were anaesthetised and given 2,000–3,000 IU of sodium heparin intravenously. Bone marrow was aspirated from the posterior iliac crests into acid citrate dextrose anticoagulant in standard blood transfusion collection packs, to give a median nucleated cell dose of $3.2 \times 10^8$ per kg recipient weight (range 1.91–4.8). The length of procedure including general anaesthetic was less than 60 min.

Harvested marrow was kept at 4°C for up to 56 h. Conditioning proceeded with melphalan 3 mg $kg^{-1}$ body weight by intravenous infusion immediately after drug reconstitution using a container protected from light. Patients receiving etoposide were given a total dose of 1,600 mg $m^{-2}$ as a continuous intravenous infusion over 20 h. TBI was given to ten patients to a total dose of 1,050 cGy in three fractions each of 350 cGy. The three fractions were given over 24 h to allow reinfusion of the unmanipulated non-cryopreserved marrow within 56 h of harvesting, using a standard blood transfusion giving set.

### *Supportive care*

Patients were nursed in single rooms and received standard supportive care during haematological recovery. All patients were given acyclovir, oral nystatin and oral amphotericin prophylactically. Multiple donor platelet transfusions were given to maintain the platelet count about $20 \times 10^9 l^{-1}$ and red cell transfusions to maintain the haemoglobin above 10 g $dl^{-1}$. All cellular blood products for transfusion were irradiated and CMV antibody negative. Three patients (2, 10, 13) received recombinant granulocyte colony-stimulating factor (rG-CSF) as part of a large multinational trial of rG-CSF therapy following ABMT.

### Results

### *Haematological reconstitution*

Neutrophil and platelet recovery occurred without delay in all patients. The rate of recovery did not vary with the differing conditioning therapies. Median number of days of neutropenia ($<0.5 \times 10^9 l^{-1}$) was 11 (range 7–20). Median number of days to a platelet count of $>50 \times 10^9 l^{-1}$ was 22 (range 13–49), but patients required transfusion of platelet concentrates on a median of only three occasions (range 1–18), and required a median of 2 units (range 0–9) of packed red cells. The conditioning regimen used did not influence neutrophil or platelet recovery times.

### *Other toxicity*

Mild oral mucositis was common (WHO grade <3). Alopecia (WHO grade 3) was universal. Nausea was minimal in patients receiving HDM and was controlled with metoclopramide. More recently, ondansetron 8 mg b.d. was used with good effect in patients receiving HDM and etoposide. The median number of days on which non-prophylactic antibiotics were given was 11 (range 0–23). There were no renal, hepatic or pulmonary complications, and no patient required intensive care. Patients stayed a median of 18 days (range 13–41) in hospital post transplant. There was no procedure-related mortality.

### *Effect on disease*

Actuarial event-free survival from the time of transplant for all patients considered for ABMT (including the two patients who relapsed prior to ABMT who were 'taken off' at '0' time) is 83% with a median follow-up of 44 months from the point of transplant (Figure 1). Only two very early relapses post ABMT, at 1 and 2 months, have occurred in the 28 patients transplanted in CR1. In addition, one patient, who had also had previous Hodgkin's disease, died 3 years post transplant from squamous carcinoma of the lung without evidence of lymphoma recurrence.

### Discussion

In high-grade NHL patients with advanced disease, the potential value of high-dose chemotherapy followed by ABMT has been reported (Phillips *et al.*, 1984; Gribben *et al.*, 1989; Petersen *et al.*, 1990). Many centres have used this form of therapy for patients in relapse or in second or third remission, and they have used very intensive conditioning regimens requiring marrow cryopreservation. Additionally, some centres have used marrow-purging techniques. A reluctance to undertake high-dose therapy with marrow rescue in first complete remission has been expressed (Gribben *et al.*, 1987) because of procedure-related mortality rates of around 20% in the larger reported series performed in advanced disease, and because of the difficulty of proving efficacy in patients in first complete remission, in whom the prognosis is more favourable than in patients with advanced disease.

The efficacy, low procedural morbidity, absent mortality and encouraging results associated with unmanipulated noncryopreserved ABMT in patients with acute lymphoblastic leukaemia (Proctor *et al.*, 1988) prompted this phase II study of ABMT in NHL patients in first CR (some of whom have already been reported; Carey *et al.*, 1991). These patients with high-grade NHL were perceived to be at high risk of relapse following conventional therapy using criteria similar to those identified by Coleman *et al.* (1986). Although the conditioning regimen was not severely toxic, it was likely to have useful activity against minimal residual disease in patients with high-grade NHL in first remission.

**Figure 1** Event-free survival of patients with high-risk, high-grade NHL referred for autologous bone marrow transplantation in first complete remission. *Patient 7 died in first complete remission at 33 months of carcinoma of the bronchus.

In this study there were no procedural deaths and the procedure-related morbidity was very low. Neutrophil and platelet recovery was more rapid than that reported using cryopreserved autologous marrow rescue (Anderson *et al.*, 1987; Hill *et al.*, 1989; To *et al.*, 1992), and neutrophil recovery (without growth factor support) was similar to rates reported with peripheral blood stem cell (PBSC) harvest techniques (Kessinger *et al.*, 1989; Bender *et al.*, 1992; Brice *et al.*, 1992; To *et al.*, 1992; Pettengell *et al.*, 1993). Platelet recovery ($>50\times10^9\,l^{-1}$) was slower than rates reported with PBSC harvest techniques (Pettengell *et al.*, 1993), but platelet transfusion requirements were similar. Unlike PBSC rescue procedures, the use of non-cryopreserved marrow has the merit of being a simple, effective and inexpensive form of rescue procedure following high-dose chemotherapy with no requirement for expert technical assistance or sophisticated equipment. The drawback to the approach is the limitation of the use of preconditioning agents with a short half-life, i.e. melphalan or etoposide, and the need to utilise TBI schedules in which delivery is complete within 24 h.

The role of first remission intensification using either marrow or PBSC rescue in intermediate and high-grade non-Hodgkin's lymphoma has been studied infrequently to date. In January 1994 the European Bone Marrow Transplant Registry (EBMT) database contained only 300 cases reported from 65 centres, including the 28 patients in the present study. In an assessment of 102 patients on the EBMT registry with Working Formulation 'high-grade' characteristics and transplanted in first remission, Sweetenham *et al.* (1994) demonstrated an overall survival of 70% and progression-free survival of 69%. This patient group had a heterogeneous

**Table II** Patient characteristics at diagnosis

| *Patient no.* | *Age* | *Sex* | *Disease histological type (Kiel)* | *Stage* | *LDH* | *Additional risk factors* | *ECOG performance status* |
|---|---|---|---|---|---|---|---|
| 1 | 46 | F | B centroblastic | IIIB | Raised | b | 3 |
| 2 | 45 | F | Centroblastic | IIIB | ND | b | 2 |
| 3 | 26 | F | Immunoblastic | IVB | ND | | 3 |
| 4 | 41 | F | Centroblastic | IB | ND | CNS disease | 3 |
| 5 | 24 | F | Lymphoblastic | IVB | ND | | 3 |
| 6 | 53 | M | T centroblastic | IIIB | Normal | | 4 |
| 7 | 58 | M | Centroblastic | IIIA | Normal | Previous HD | 3 |
| 8 | 36 | M | Centroblastic | IIB | ND | b | 3 |
| 9 | 25 | M | Centroblastic | IIB | ND | b | 4 |
| 10 | 41 | M | Centroblastic | IVB | ND | b | 3 |
| 11 | 54 | M | Centroblastic | IIIB | ND | b | 3 |
| 12 | 38 | M | Centroblastic | IIIA | Normal | b | 2 |
| 13 | 21 | M | Immunoblastic | IVB | ND | | 3 |
| 14 | 44 | F | Centroblastic | IIIA | ND | BM | 4 |
| 15 | 29 | M | Centroblastic | IIIB | ND | b | 4 |
| 16 | 30 | M | T lymphoblastic | IVB | Raised | BM | 2 |
| 17 | 50 | M | B lymphoblastic | IIIB | ND | | 2 |
| 18 | 26 | M | B lymphoblastic | IVA | ND | | 2 |
| 19 | 33 | F | B lymphoblastic | IVB | ND | | 4 |
| 20 | 38 | F | T lymphoblastic | IVA | ND | | 2 |
| 21 | 16 | M | B lymphoblastic | IIA | Raised | b | 3 |
| 22 | 39 | M | B lymphoblastic | IVA | ND | | 2 |
| 23 | 39 | M | T lymphoblastic | IVA | ND | | 3 |
| 24 | 49 | M | Centroblastic | IVA | ND | | 3 |
| 25 | 41 | M | Centroblastic | IVB | ND | b | 2 |
| 26 | 34 | M | Centroblastic | IIB | Raised | b | 2 |
| 27 | 32 | F | T immunoblastic | IIIA | ND | b | 4 |
| 28 | 30 | M | Centroblastic | IIIB | Raised | b | 2 |
| 29 | 27 | M | B lymphoblastic | IVB | } Relapsed prior to planned BMT | | |
| 30 | 24 | M | B lymphoblastic | IVB | | | |

All lymphoblastic non-Burkitt type. ND, not done; BM, bone marrow disease; b, bulky disease; T, T cell; B, B cell. All patients in this series fulfilled the Kiel histological classification of high grade non-Hodgkin's lymphoma, which includes diffuse centroblastic disease. The 16 patients with centroblastic disease within the Working Formulation classification would be considered intermediate grade.

form of preconditioning, nevertheless the trend of improved event-free survival for such difficult patients remains encouraging. Other single-centre studies with similar patient populations to the present study have been performed utilising more ablative chemotherapy regimens and marrow purging in some studies (Philip et al., 1988; Colombat et al., 1990; Nademanee et al., 1992; Freedman et al., 1993). All these studies have provided encouraging data and included substantial proportions of intermediate NHL according to the Working Formulation as in the present study. The Stanford study on 20 patients reports an 84% disease-free survival at 3 years when transplanted in first CR (Nademanee et al., 1992). Philip et al. (1988) and a series from the Dana Faber Cancer Centre (Freedman et al., 1993) reported similar promising results. The only substantial trial of autologous transplant in first CR versus intensive chemotherapy is that performed by the French National Lymphoma Group, and preliminary data presented at the American Society of Haematology Meeting in December 1993 (Haioun et al., 1993) indicated that in a formal trial setting well-delivered aggressive chemotherapy consolidation is equivalent to autotransplant intensification in first CR.

With a median follow-up of nearly 4 years following transplant, the event-free survival of 83% in this group of 'high-risk', high-grade NHL patients considered for ABMT in first complete remission suggests that this may be a useful consolidation therapy following remission induction chemo/radiotherapy. Recently a number of indices have been developed to delineate poor-prognosis groups in patients with NHL (Hayward et al., 1991; The International Non-Hodgkin's Lymphoma Prognostic Factors Project, 1993). One index has been developed and validated by the SNLG (Hayward et al., 1991) on patients treated with the same remission induction chemotherapy as in this study. This analysis highlighted age, stage, performance status, abnormal white cell count, extranodal disease and B symptoms as risk factors. In our group of patients, seven were low risk, six were intermediate risk and 17 were high risk with predicted 5 year survivals of 54%, 31% and 11% respectively. It should be noted that our patients in the low/intermediate prognostic groups had either stage IV or bulk disease, regarded by other groups as independent indicators of poor prognosis.

As this study commenced 9 years ago the lactate dehydrogenase, a universally accepted and powerful prognostic factor, was not always measured at diagnosis (see Table II). Despite not always being able to add this additional risk factor, 22 of our patients were in the high and high-intermediate risk groups with the remaining eight patients in the low-intermediate prognostic group according to the International Non-Hodgkin's Lymphoma Prognostic Factors Project (The International Non-Hodgkin's Lymphoma Prognostic Factors Project, 1993). The 5 year relapse-free survival rates after achieving complete remission for these groups according to the project were 46%, 32% and 66% respectively.

We have shown that ABMT without cryopreservation is a safe procedure which merits further study in patients with high-risk, high-grade NHL in first complete remission and, as a result of this pilot information, a prospective randomised controlled study comparing ABMT with no consolidation therapy in this group of patients is currently in progress under the auspices of the SNLG. Similar studies in patients in first remission are planned by groups in Europe using more intensive conditioning regimens such as BEAM (BCNU, etoposide, cytosine arabinoside and melphalan). The well-documented mortality and toxicity of these regimens have given rise to misgivings about such an approach being ethical in first complete remission. Our data suggest that a good clinical outcome can be seen following less aggressive intensification. Apart from the two early relapses (2 months post ABMT) no patients undergoing this form of intensification have relapsed. The paucity of late relapses in this study provides strong evidence that more ablative and therefore more toxic regimens may not be necessary to condition patients with poor-prognosis high-grade NHL in CR1 for ABMT.

If the promising results of this phase II study are borne out in the prospective randomised study, this may well point the way for a more effective and less toxic treatment of patients with high-risk, high-grade, non-Hodgkin's lymphoma.

**Note** Individual lymphoma groups interested in possible collaboration utilising an approach of intensification as part of a randomised trial of the Scotland and Newcastle Lymphoma Group should contact Professor S.J. Proctor, Department of Haematology, Royal Victoria Infirmary, Queen Victoria Road, Newcastle upon Tyne, NE1 4LP (Tel: 091 232 5131 ext. 24261, Fax 091 230 0651).

The authors would like to thank Dr M.M. Reid for his helpful reading of the manuscript and Mrs Margaret Graham for her excellent secretarial support.

### References

ANDERSON, C.C., GOLDSTONE, A.H., LINCH, D.C., JONES, H.M., FRANKLIN, I.M., BOUGHTON, B.J., CAWLEY, J.C. & RICHARDS, J.D.M. (1987). Autologous bone marrow transplantation for patients with acute myeloid leukaemia and acute lymphoblastic leukaemia – a comparison. *Bone Marrow Transplant*, **1**, 271–279.

ARMITAGE, J.O., WEISENBURGER, D.D., HUTCHINS, M., MORAVEC, D.F., DOWLING, M., SORENSEN, S., MAILLIARD, J., OVERBLOOM, J., JOHNSON, P.W. & HOWE, D. (1986). Chemotherapy for diffuse large-cell lymphoma – rapidly responding patients have more durable remissions. *J. Clin. Oncol.*, **4**, 160–164.

BENDER, J.G., WILLIAMS, S.F., MYERS, S., NOTTLEMAN, D., LEE, W.J., UNVERZAGT, K.L., WALKER, D., TO, L.B. & VAN EPPS, D.E. (1992). Characterization of chemotherapy mobilized peripheral blood progenitor cells for use in autologous stem cell transplantation. *Bone Marrow Transplant*, **10**, 281–285.

BRICE, P., MAROLLEAU, J.P., DOMBRET, H., LEPAGE, E., BARUCHEL, A., ADAM, M., MICLEA, J.M., SITTHY, X. & GISSELBRECHT, C. (1992). Autologous peripheral blood stem cell transplantation after high dose therapy in patients with advanced lymphomas. *Bone Marrow Transplant.*, **9**, 337–342.

CAREY, P., PROCTOR, S.J., TAYLOR, P., HAMILTON, P.J. ON BEHALF OF THE NORTHERN REGIONAL BONE MARROW TRANSPLANT GROUP (1991). Autologous bone marrow transplantation for high grade lymphoid malignancy using melphalan/irradiation conditioning without marrow purging or cyropreservation. *Blood*, **77**, 1593–1598.

COIFFIER, B., GISSELBRECHT, C., HERBRECHT, R., TILLY, H., BOSLM, A. & BROUSSE, N. (1989). LNH-84 regimen: a multicentre study of intensive chemotherapy in 737 patients with aggressive malignant lymphoma. *J. Clin. Oncol.*, **7**, 1018–1026.

COLEMAN, C.N., PICOZZI, V.J., COX, R.S., MCWHIRTER, K., WEISS, L.M., COHEN, J.R., YU, K.P. & ROSENBERG, S.A. (1986). Treatment of lymphoblastic lymphoma in adults. *J. Clin. Oncol.*, **4**, 1628–1637.

COLOMBAT, P., GORIN, N.G., LEMONNIER, M.P. & 7 others (1990). The role of autologous bone marrow transplantation in 46 adult patients with non-Hodgkin's lymphomas. *J. Clin. Oncol.*, **8**, 630–637.

FREEDMAN, A.S., TAKVORIAN, T., NEUBERG, D. & 12 others (1993). Autologous bone marrow transplantation in poor prognosis intermediate-grade and high-grade B-cell non-Hodgkin's lymphoma in first remission: a pilot study. *J. Clin. Oncol.*, **11**, 931–936.

GRIBBEN, J.G., VAUGHAN HUDSON, B. & LINCH, D.C. (1987). The potential value of very intensive therapy with autologous bone marrow rescue in the treatment of malignant lymphomas. *Hematol. Oncol.*, **5**, 281–286.

GRIBBEN, J.G., GOLDSTONE, A.H., LINCH, D.C., TAGHIPOUR, G., MCMILLAN, A.K., SOUHAMI, R.L., EARL, H. & RICHARDS, J.D. (1989). Effectiveness of high dose combination chemotherapy and autologous bone marrow transplantation for patients with non-Hodgkin's lymphoma who are still responsive to conventional dose chemotherapy. *J. Clin. Oncol.*, **7**, 1621–1629.

HAIOUN, C., LEPAGE, E., GISSELBRECHT, C., COIFFIER, B., BOSLY, A., TILLY, H., MOREL, P., NOUVEL, C., HERBRECHT, R., D'AGAY, M.F., GAULARD, P. & REYES, F. (1993). Comparison of autologous bone marrow transplantation (ABMT) with sequential chemotherapy for aggressive non-Hodgkin's lymphoma (NHL) in first complete remission: a study on 464 patients (LNH87 protocol). *Blood*, **82** (Suppl. 1), 87a.

HAYWARD, R.L., LEONARD, R.C.F., PRESCOTT, R.J., ALLAN, N.C., DAS, S.N., DAWSON, A.A., HEPPLESTON, A., LESSELLS, A.M., LUCRAFT, H.H., MACKIE, M.J., MACGILLIVRAY, J.B., MACLAREN, K.S., PARKER, A.C., PROCTOR, S.J., RITCHIE, G.L., SARKAR, T.K. & WHITE, J.M. (1991). A critical analysis of prognostic factors for survival in intermediate and high grade non-Hodgkin's lymphoma. *Br. J. Cancer*, **63**, 945-952.

HILL, R.S., MAZZA, P., AMOS, D., BUCKNER, C.D., APPELBAUM, F.R., MARTIN, P., STILL, B.J., SICA, S., BERENSON, R., BENSINGER, W., CLIFT, R.A., STOWART, P., DONEY, K., SANDERS, J., SINGER, J., SULLIVAN, K.M., WITHERSPOON, R.P., STORB, R., LIVINGSTON, R., CHARD, R. & THOMAS, E.D. (1989). Engraftment in 86 patients with lymphoid malignancy after autologous marrow transplantation. *Bone Marrow Transplant*, **4**, 69-74.

KESSINGER, A., ARMITAGE, J.O., SMITH, D.M., LANDMARK, J.D., BIERMAN, P.J. & WEISSENBURGER, D.D. (1989). High-dose therapy and autologous peripheral blood stem cell transplantation for patients with lymphoma. *Blood*, **74**, 1260-1265.

LEONARD, R.C.F., PRESCOTT, R.J., MAO, J.-H., WHITE, J.M., WITH MEMBERS OF THE SNLG THERAPY WORKING PARTY AND PATHOLOGY WORKING PARTY: ALLAN, N.C., BARRETT, A., DAS, S.N., DAWSON, A.A., HEPPLESTON, A., LENNARD, A., LESSELLS, A.M., LUCIE, N.P., LUCRAFT, H.H., MACKIE, M.J., MACLAREN, K.S., MATHESON, L.M., PARKER, A.C., PROCTOR, S.J., ROBERTSON, A.G., SARKAR, T.K., SOUKOP, M., STEWARD, W.P., TANSEY, P., ANGUS, B., HORNE, C.H.W., KRAJEWSKI, A.S., MACGILLIVRAY, J.B. & THOMPSON, W.D. (1993). Successful application of a previously derived prognostic index in the analysis of a randomised trial of 281 patients with high grade non-Hodgkin's lymphoma (HIGNHL). *Ann. Oncol.*, **4**, 853-856.

NADEMANEE, A., SCHMIDT, G.M., O'DONNELL, M.R. & 11 others (1992). High-dose chemoradiotherapy followed by autologous bone marrow transplantation as consolidation therapy during first complete remission in adult patients with poor risk aggressive lymphoma. *Blood*, **80**, 1130-1134.

PETERSEN, F.B., APPELBAUM, F.R., HILL, R., FISHER, L.D., BIGELOW, C.L., SANDERS, J.E., SULLIVAN, K.M., BESINGER, W.I., WITHERSPOON, R.P., STORB, R., CLIFT, R.A., FEFER, A., PRESS, O.W., WEIDEN, P.L., SINGER, J., THOMAS, E.D. & BUCKNER, C.D. (1990). Autologous marrow transplantation for malignant lymphoma: a report of 101 cases from Seattle. *J. Clin. Oncol.*, **8**, 638-647.

PETTENGELL, R., MORGENSTERN, G.R., WOLL, P.J., CHANG, J., ROWLANDS, M., YOUNG, R., RADFORD, J.A., SCARFFE, J.H., TESTA, N.G. & CROWTHER, D. (1993). Peripheral blood progenitor cell transplantation in lymphoma and leukemia using a single apheresis. *Blood*, **82**, 3770-3777.

PHILIP, T., HARTMANN, O., BIRON, P., CAHN, J.Y., PEIN, F., BORDIGONI, P., SOUILLET, G., LASSET, C. & CHAUVIN, F. (1988). High dose therapy and autologous bone marrow transplantation in partial remission after first line induction therapy for diffuse non-Hodgkin's lymphoma. *J. Clin. Oncol.*, **6**, 1118-1124.

PHILLIPS, G.L., HERZIG, R.H., LAZARUS, H.M., FAY, J.W., WOLFF, S.N., MILL, W.B., LIN, H.S., THOMAS, P.R.M., GLASGOW, G.P., SHINA, D.C. & HERZIG, G.P. (1984). Treatment of resistant malignant lymphoma with cyclosphosphamide, total body irradiation, and transplantation of cryopreserved autologous marrow. *N. Engl. J. Med.*, **310**, 1557-1561.

PROCTOR, S.J., HAMILTON, P.J., TAYLOR, P., CAREY, P., HARGRAVE, S., EVANS, R.G.B., SUMMERFIELD, G., FINNEY, R., SAUNDERS, P., GOFF, D. & REID, M.M. (1988). A comparative study of combination chemotherapy versus marrow transplant in first remission in adult acute lymphoblastic leukaemia. *Br. J. Haematol.*, **69**, 35-39.

SKARIN, A.T. (1986). Diffuse aggressive lymphomas: a curable subset of non-Hodgkin's lymphoma. *Semin. Oncol.*, **13**, 10-25.

SWEETENHAM, J.W., PROCTOR, S.J., BLAISE, D., LAURENZI, A., PEARCE, R., TAGHIPOUR, G., GOLDSTONE, A.H. FOR THE LYMPHOMA WORKING PARTY OF THE EUROPEAN GROUP FOR BONE MARROW TRANSPLANTATION (1994). High dose therapy and autologous bone marrow transplantation (ABMT) in first complete remission for adult patients with high grade non-Hodgkin's lymphoma: the EBMT experience. *Ann. Oncol.*, **5**, S155-S159.

TAYLOR, P.R.A., JACKSON, G.H., LENNARD, A.L., LUCRAFT, H., PROCTOR, S.J. ON BEHALF OF THE NEWCASTLE AND NORTHERN REGION LYMPHOMA GROUP (1993). Autologous transplantation in poor risk Hodgkin's disease using high dose melphalan/etoposide conditioning with non-cryopreserved marrow rescue. *Br. J. Cancer*, **67**, 383-387.

THE INTERNATIONAL NON-HODGKIN'S LYMPHOMA PROGNOSTIC FACTORS PROJECT (1993). A predictive model for aggressive non-Hodgkin's lymphoma. *N. Engl. J. Med.*, **329**, 987-994.

TO, L.B., ROBERTS, M.M., HAYLOCK, D.N., DYSON, P.G., BRANFORD, A.L., THORP, D., HO, J.Q.K., DART, G.W., HORVATH, N., DAVY, M.L.J., OLWENY, C.L.M., ABDI, E. & JUTTNER, C.A. (1992). Comparison of haematological recovery times and supportive care requirements of autologous recovery phase peripheral blood stem cell transplants, autologous bone marrow transplants and allogeneic bone marrow transplants. *Bone Marrow Transplant.*, **9**, 277-284.